# Polyethylene glycol modified LAMP assay enables sensitive and specific clinical detection of varicella-zoster virus

**DOI:** 10.3389/fcimb.2025.1718986

**Published:** 2025-12-09

**Authors:** Lei Zhang, Jun Tian, Wenjie Fang, Bin Xu, Weinan Guo, Saiping Lv, Li Yang

**Affiliations:** 1 Department of Dermatology and Venerology, Shaanxi Provincial People’s Hospital, Xi’an, China; 2 Department of Dermatology, Xijing Hospital, Fourth Military Medical University, Xi’an, Shaanxi, China; 3 Department of Dermatology, Shanghai Key Laboratory of Molecular Medical Mycology, The Center for Basic Research and Innovation of Medicine and Pharmacy (MOE) Shanghai Changzheng Hospital, Naval Medical University, Shanghai, China; 4 Jiangxi Key Laboratory of Oncology, Jiangxi Cancer Hospital & Institute, The Second Affiliated Hospital of Nanchang Medical College, Nanchang, Jiangxi, China; 5 JXHC Key Laboratory of Tumor Metastasis, Jiangxi Cancer Hospital & Institute, The Second Affiliated Hospital of Nanchang Medical College, Nanchang, Jiangxi, China; 6 Department of Medical Laboratory, Jiangxi Cancer Hospital & Institute, Nanchang, Jiangxi, China

**Keywords:** varicella-zoster virus, diagnosis, molecular diagnosis, LAMP assay, infectious disease

## Abstract

Accurate, timely diagnosis of varicella–zoster virus (VZV) is important for treatment and infection control. While loop-mediated isothermal amplification (LAMP) is operationally simple, nonspecific priming can degrade performance. We assessed a polyethylene glycol–modified LAMP (PEG-LAMP) that tunes the reaction microenvironment via macromolecular crowding. PEG (1 μL per reaction) was titrated across concentrations; 100 mM was selected as the optimized condition because negatives remained at baseline while target amplification kinetics were maintained. PEG-LAMP preserved a log–linear relation between threshold time and input and improved the detection limit from 10^3^ to 10^2^ copies/μL compared with conventional LAMP. Precision at a fixed input exhibited low variability, and specificity was supported by flat traces in non-target reactions. In a 30-sample panel (15 spiked positives, 15 negatives) tested in parallel by PCR, conventional LAMP, and PEG-LAMP, PEG-LAMP was fully concordant with PCR and yielded shorter time-to-threshold for positives, whereas conventional LAMP produced one false negative and four false positives. Taken together, the results demonstrate that microenvironmental tuning with PEG provides a low-complexity means to suppress nonspecific LAMP while preserving on-target amplification, yielding a lower detection limit and faster time-to-result with PCR-level qualitative agreement in clinical VZV diagnosis.

## Introduction

Varicella-zoster virus (VZV), one of human herpes viruses, primarily causes varicella (chickenpox) and can later reactivate to cause herpes zoster (HZ) either spontaneously or after several triggering factors (Kennedy and Gershon, 2018). The lifetime risk of developing either varicella or HZ is as high as 32%, and the incidence of HZ increases with age (Wutzler et al., 2017). A recent systematic review showed an incidence rate of HZ ranging from 5.23 to 10.9 cases per 1,000 person-years (van Oorschot et al., 2021). HZ may lead to serious complications, including post-herpetic neuralgia (PHN), HZ ophthalmicus, neurological complications, and even disseminated diseases in immunocompromised individuals (Lim et al., 2024).

The diagnosis of VZV infection is typically clinical, based on the presence of characteristic unilateral clusters of papulovesicular lesions on an erythematous base that do not cross the midline (Dayan and Peleg, 2017). However, when it comes to atypical cutaneous manifestations which is common in immunocompromised patients or aged patients, the diagnosis would be vital challenging, especially for non-specialist including primary care or emergency settings, and delayed treatment are usually associated with substantial economic burden and impairment of life quality (Daruish et al., 2024; Scampoli et al., 2024). In these context, additional tests may be required such as biopsies, serology tests and PCR (John and Canaday, 2017). These current laboratory tests are not always feasible as biopsy is invasive, time consuming and limited sensitivity; serology tests is subjected to host immune status and is limited sensitivity in early diagnosis (Otani et al., 2020). Nucleic-acid amplification tests based on real-time PCR are widely regarded as the reference for clinical diagnosis (Inata et al., 2018), but they require thermocycling instruments, stable power, and trained personnel (Gross et al., 2020). In settings with lower throughput or limited equipment, laboratories often rely on methods that trade turnaround time for simplicity. There remains a need for assays that couple rigorous analytical performance with simplified workflows.

Loop-mediated isothermal amplification (LAMP) is attractive in this context because it runs at a constant temperature, tolerates some matrix effects, and can be read in real time or by simple endpoints (Notomi et al., 2015). However, the method is prone to nonspecific priming due to the use of multiple high concentration primers and the formation of secondary structures (Kim et al., 2023). This can lead to late-rising background in negative samples and, in some designs, occasional false positives. As a result, many LAMP implementations add probes (Shen et al., 2024), adjust primer sets (Ye et al., 2018; Ye et al., 2019), or apply strict interpretation rules, which complicate otherwise simple workflows.

An alternative route is to modify the reaction microenvironment to favor correct primer–template pairing at the earliest stages of amplification. Macromolecular crowding reagents such as polyethylene glycol (PEG) reduce water activity and impose excluded-volume effects that change the stability landscape of nucleic-acid complexes (Qin et al., 2017; Yu et al., 2023; Yang et al., 2024). In principle, this can stabilize fully matched duplexes while reducing mismatches and primer–primer contacts, suppressing the initiation events that trigger nonspecific amplification. If achievable without inhibiting polymerase function or strand displacement, this strategy could improve LAMP specificity and preserve or enhance sensitivity without redesigning primers or adding probes.

Here, we evaluate a polyethylene glycol modified LAMP (PEG-LAMP) assay for the clinical detection of varicella–zoster virus (VZV). We ask whether a small PEG addition can suppress background in negatives, preserve efficient amplification in positives, and thereby improving diagnostic accuracy relative to conventional LAMP while keeping the workflow aligned with routine practice. We discuss the underlying mechanism, define an optimized condition that balances specificity and kinetics, and conduct a comparative assessment of PEG-LAMP versus conventional LAMP and PCR on a mixed positive and negative specimen panel. In clinical use, this simple modification can reduce false positives without altering primers or instrumentation, shorten time to result, and enable earlier antiviral decisions and infection control; in resource-limited settings, it may serve as a practical alternative or complement to PCR.

## Materials and methods

### Specimens and nucleic acid preparation

The varicella-zoster virus DNA standard (BNCC364708) was purchased form BeNa culture collection. The matrices (vesicular fluid) were transported at 2–8 °C in viral transport media and stored at −80 °C. Spiked positives were prepared by adding a quantified VZV nucleic-acid standard to clinical matrices before extraction and processed alongside native negatives. Nucleic acids were extracted with a commercial column virus DNA purification kit (B518267) purchased from Shanghai Sangon Biotech. Each run included a quantified VZV positive control and a no-template control (ultrapure water, NTC).

### Assay reagents and conditions

Each LAMP reaction was assembled on ice in a final volume of 25 µL and contained 1× isothermal buffer, MgSO_4_ 6–8 mM, dNTPs 1.4 mM each, and strand-displacing DNA polymerase (Bst 2.0 Warmstart; 8 U per reaction). Primers targeting VZV were added at standard LAMP ratios (final: FIP/BIP 1.6 µM, LF/LB 0.4 µM, F3/B3 0.2 µM; sequences in **Table 1**). Template input was ~2 µL of extracted nucleic acid. For PEG-modified reactions, 1 µL of PEG stock was added at the indicated concentrations (0, 25, 50, 100, 200, or 500 mM); no-PEG controls received 1 µL nuclease-free water to maintain volume. Reactions were briefly spun down, sealed, and incubated isothermally for up to 60 min with real-time fluorescence acquisition.

**Table 1:** The LAMP primers targeting the VZV used in this study.

Item	Sequences
F3	CGACGGGTGTCTCCCTAA
B3	GGCAACGGGCTCCAGT
FIP	TGTGTCCACCGGATGATCGTTTGAGGCTTCTGCTCTCGACTG
BIP	CAGAAAGAGAGCGTGCGGCGGCGGTCACCCTTCTCCAAC
LF	ACGAACTCCGCGCAAGC
LB	GAGGGTCGGGAGTCTGTGT

The primer sequences were checked by in-silico BLAST against the NCBI nucleotide database to identify any potential off-target matches. We also examined a minimal cross-reactivity panel that included closely related species and common background organisms to confirm that the primers did not produce nonspecific amplification.

### Experimental evaluations

The PEG solution was purchased from The Sigma-Aldrich brand. At a fixed addition volume of 1 μL, PEG stock concentrations of 0, 25, 50, 100, 200, and 500 mM were evaluated. For each concentration, four VZV-positive and four VZV-negative specimens were run in parallel under identical conditions. The concentration offering the best balance between background suppression and target amplification was subsequently verified using twelve independent positives.

Ten-fold VZV DNA dilutions (10^7–^10^1^ copies/μL) were tested with and without PEG in parallel. Threshold time (Ct, min) was plotted against log10(input) and fitted by linear regression to assess log-linear behavior. The limit of detection (LoD) was defined as the lowest concentration detected within 60 min. Specificity was assessed using multiple VZV-negative specimens and stability was determined from n=24 technical replicates at a fixed input and summarized as the coefficient of variation of Ct.

A 30-sample panel was analyzed in parallel by PCR, conventional LAMP, and PEG-LAMP. Samples 1–15 were spiked positives prepared by adding quantified VZV DNA standard to negative clinical matrices before extraction; samples 16–30 were negatives. For each method, per-sample Ct and qualitative calls were recorded. Using PCR as the reference, sensitivity, specificity, and overall agreement were calculated.

## Results

### Polyethylene glycol suppresses nonspecific priming in LAMP by enforcing correct primer–template pairing

**Figure 1:**
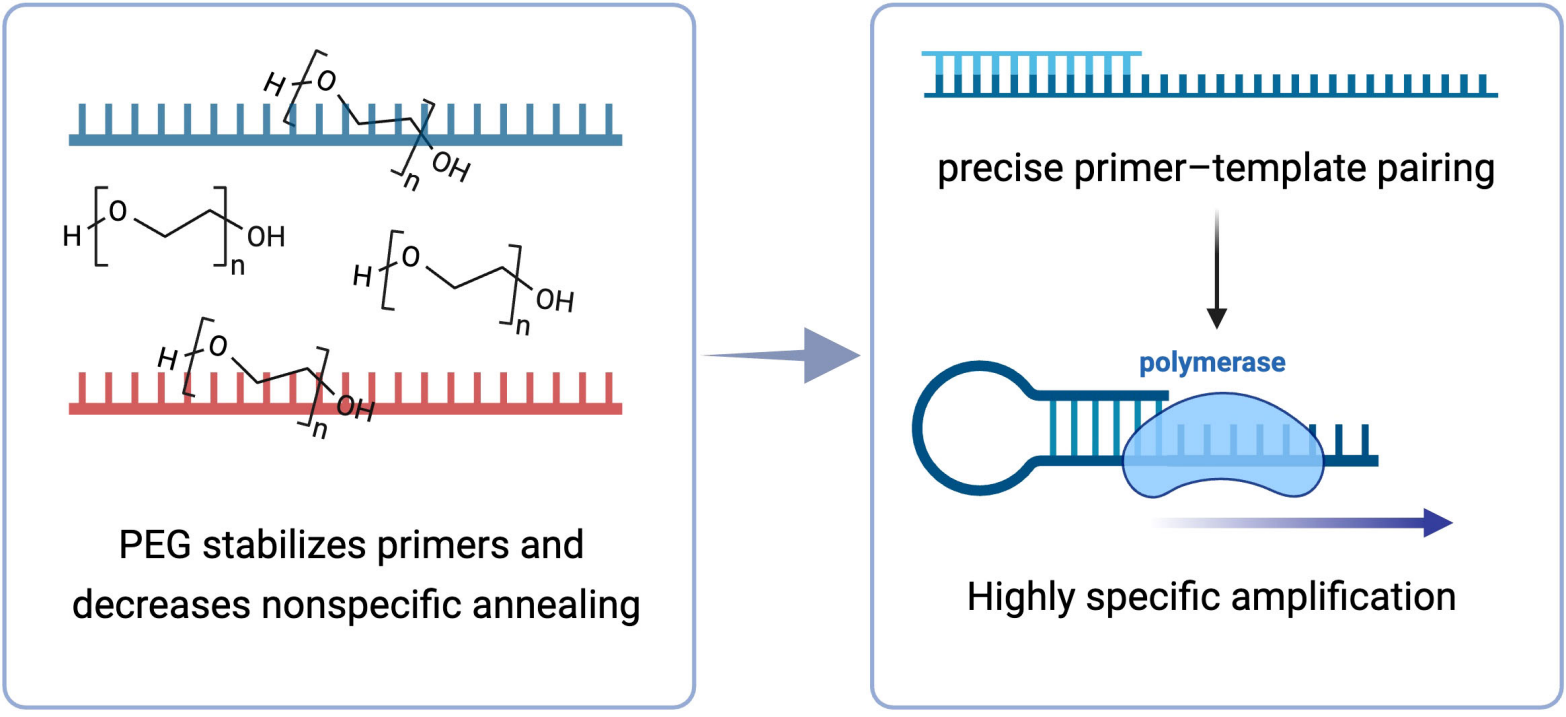
Polyethylene glycol improves LAMP specificity by stabilizing correct primer–template pairing and suppressing nonspecific annealing.

A longstanding limitation of loop-mediated isothermal amplification (LAMP) is the accumulation of background products arising from primer–primer interactions and mismatched annealing. Introducing polyethylene glycol (PEG) into the reaction mixture reduced these events and sharpened assay readouts. As summarized in **Figure 1**, PEG suppressed off-target hybridization events while in preference to stabilizing fully matched primer–template duplexes.

Mechanistically, PEG acts as a macromolecular crowding agent that lowers water activity and imposes excluded-volume constraints around oligonucleotides. This microenvironment elevates the effective concentration of reactants and, relative to fully matched duplexes, increases the thermodynamic restrictive associated with imperfect hybrids. Consequently, pairing equilibria are shifted toward correct primer–template complexes and away from transient nonspecific or primer–primer dimers. Stabilization of the correct initiation complex facilitates productive strand displacement by the polymerase and efficient formation of classic LAMP loop intermediates, thereby directing reagents into the on-target amplification pathway while suppressing off-target branches (**Figure 1**). The same trend—lower background and improved target amplification—enhanced the higher diagnostic specificity and sensitivity observed in our clinical Varicella-Zoster Virus (VZV) assays reported below.

### The optimized PEG concentration enables LAMP specificity without compromising amplification of VZV-positive samples

**Figure 2:**
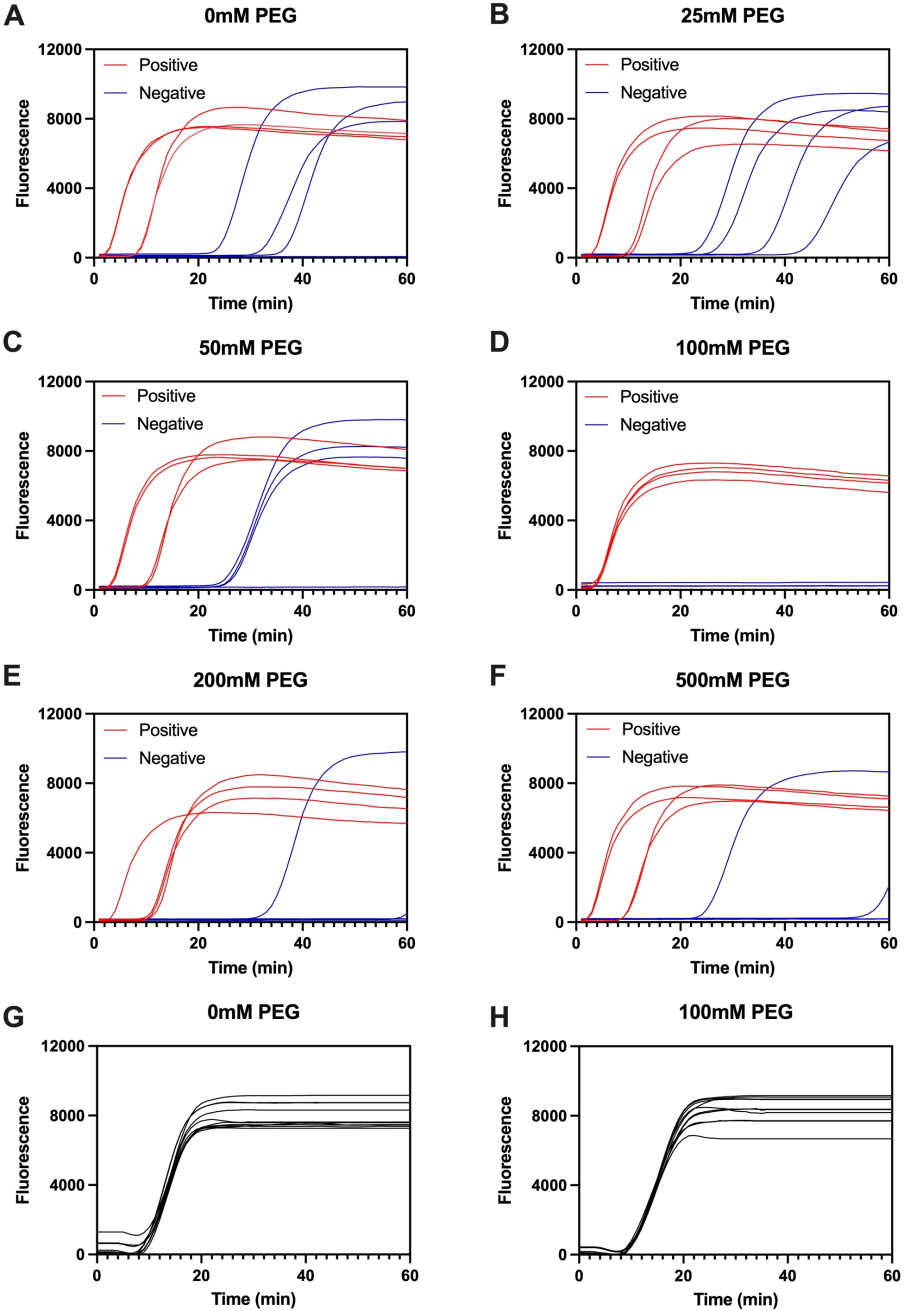
PEG titration identifies conditions that suppress nonspecific LAMP while maintaining robust amplification of VZV-positive samples. **(A–F)** Real-time fluorescence curves for LAMP reactions containing 1 μL PEG at different concentrations: **(A)** 0 mM, **(B)** 25 mM, **(C)** 50 mM, **(D)** 100 mM, **(E)** 200 mM, and **(F)** 500 mM. Each panel shows four VZV-positive samples (red) and four VZV-negative samples (blue). Verification of the optimal condition using an expanded set of twelve VZV-positive specimens with 0 mM PEG **(G)** and 100 mM PEG **(H)**.

To define working conditions for PEG-mediated suppression of nonspecific LAMP products, we titrated PEG (1 μL added per reaction) across six concentrations and monitored amplification of four Varicella–Zoster virus (VZV)-positive and four VZV-negative clinical specimens in each condition (**Figures 2A–F**). In the absence of PEG, negative reactions exhibited late-rising background traces characteristic of nonspecific LAMP byproducts, while positives showed typical sigmoidal kinetics (**Figure 2A**). Introducing low-to-moderate PEG (25–50 mM) visibly reduced the frequency and amplitude of background elevation in negatives (**Figures 2B, C**). At 100 mM PEG, negative reactions remained flat throughout the assay process, and positive reactions retained steep rises and stable plateaus (**Figure 2D**). This condition provided the clearest discrimination between positives and negatives, indicating effective suppression of spurious priming while preserving on-target amplification. At higher PEG (200–500 mM), suppression of background persisted; however, positive curves displayed broader variability in rise profiles (**Figures 2E, F**), consistent with the expectation that excessive macromolecular crowding can begin to affect polymerase-driven strand displacement. Collectively, these titrations identify 100 mM PEG (1 μL) as an optimized amount that maximizes specificity without imposing a kinetic suppression on target amplification.

We then assessed generalizability at this condition using an expanded cohort of twelve VZV-positive specimens. All twelve amplified robustly with 100 mM PEG, with kinetics comparable to or sharper than the no-PEG control set (**Figures 2G, H**). Thus, adding 1 μL of 100 mM PEG not only suppresses nonspecific signals in negatives but also does not inhibit amplification in true positives, thereby expanding the diagnostic application for clinical VZV detection.

### Analytical performance of PEG-LAMP: dynamic range, specificity, and stability

**Figure 3:**
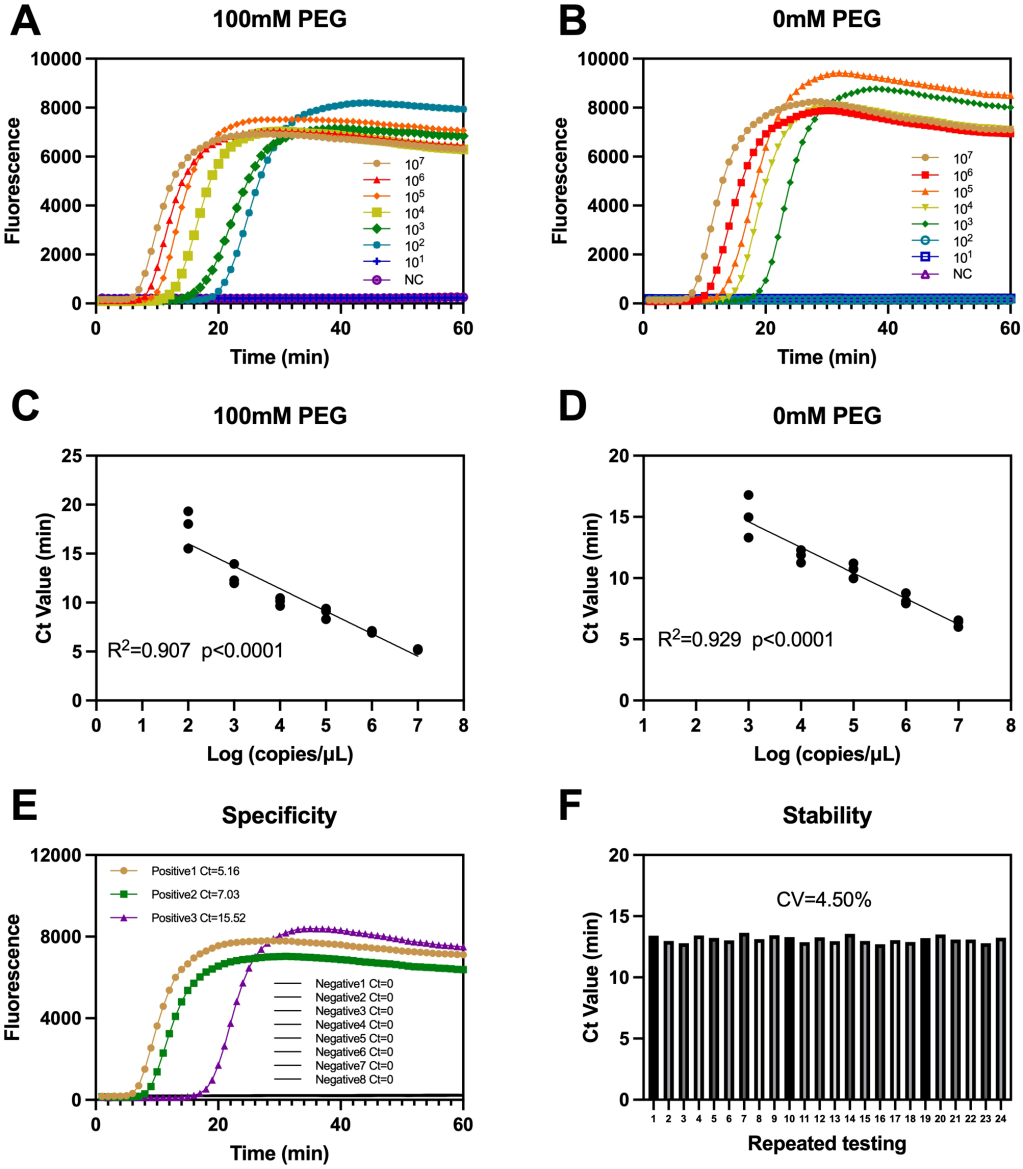
Analytical characteristics and sensitivity of the PEG-modified LAMP assay. Dilution series (10^7–^10^1^ copies/μL) with 100 mM PEG (1 μL) **(A)** and No PEG **(B)**. Standard curves of Ct (min) versus log10(input) with PEG **(C)** and without PEG **(D)**. **(E)** Specificity under the PEG condition. **(F)** Stability under the PEG condition across 24 replicates.

Serial dilutions of VZV DNA (10^7–^10^1^ copies/μL) generated orderly sigmoidal curves under the PEG condition, with negatives remaining at baseline (**Figure 3A**). The no-PEG control amplified across the series but showed slower kinetics at low input and a narrower separation among adjacent dilutions (**Figure 3B**). Threshold time (Ct, min) correlated with input in both settings (**Figures 3C, D**). Importantly, the limit of detection (LoD)—defined as the lowest dilution consistently detected within the run time—reached 10^2^ copies/μL with 100 mM PEG (1 μL), whereas the no-PEG assay detected down to 10^3^ copies/μL under otherwise identical conditions. Thus, PEG improved analytical sensitivity by one order of magnitude while preserving the monotonic log–linear response.

Specificity testing under the PEG condition showed flat curves for all VZV-negative specimens, while representative positives yielded Ct values of 5.16, 7.03, and 15.52 min (**Figure 3E**). Repeat testing (n = 24) produced a Ct coefficient of variation of 4.50% (**Figure 3F**), indicating stable performance. Together, these data show that adding 1 μL of 100 mM PEG maintains a log-linear dilution response, improves separation of true positives from background, and yields low run-to-run variability.

### Clinical validation and method comparison

**Figure 4:**
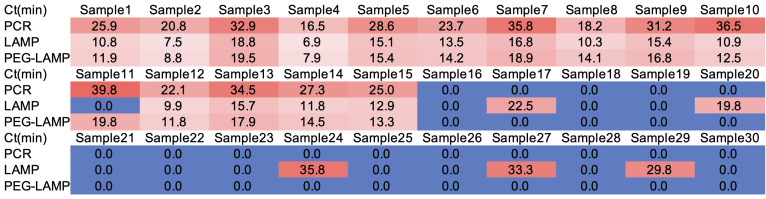
Per-sample Ct (min) across PCR, LAMP, and PEG-LAMP on a 30-sample panel. Heatmap of threshold times for Samples 1–15 (spiked positives) and Samples 16–30 (negatives). Rows show PCR (reference), LAMP, and PEG-LAMP (100 mM PEG, 1 μL).

We compared PEG-LAMP with conventional LAMP using a 30-sample panel (Samples 1–15 spiked positives; 16–30 negatives) with PCR as the reference (**Figure 4**). PEG-LAMP was fully concordant with PCR: all 15 positives and all 15 negatives were correctly classified. Across positives, PEG-LAMP yielded consistently shorter threshold times than PCR, indicating faster target detection without loss of accuracy.

Conventional LAMP showed one false negative (Sample 11) among the positives and four false positives (Samples 17, 20, 24, 29) among the negatives. Relative to PCR on this panel, this corresponds to a sensitivity of 14/15 and a specificity of 11/15. PEG-LAMP therefore removed the false positives observed with LAMP and recovered the missed positive, while maintaining earlier thresholds than PCR.

## Discussion

The present work addresses a long-standing constraint of LAMP—nonspecific priming—by modifying the reaction microenvironment rather than redesigning primers or adding probe strategies (Gadkar et al., 2018; Yang et al., 2023). Introducing a small amount of polyethylene glycol (PEG) shifted the balance of early hybridization events toward correct primer–template pairing and away from primer–primer or mismatched interactions. This interpretation is consistent with a macromolecular crowding mechanism: reduced water activity and excluded-volume effects increase the effective concentration of reactants and reduce the mismatched duplex formation. Under these conditions, initiation proceeds more efficiently into the canonical looped intermediates, yielding lower baseline variability and more reproducible amplification kinetics.

A practical outcome of this adjustment is a wider diagnostic application: negatives showed no rise above baseline, while positives amplified with early thresholds, and the detection limit improves by one order of magnitude compared with conventional LAMP (Okamoto et al., 2004; Kaneko et al., 2005). Importantly, these gains do not require changes to the primer set or the temperature program, which facilitates adoption in laboratories already operating LAMP workflows. The comparative panel indicates that PEG-LAMP achieves qualitative agreement comparable to PCR while shortening time-to-result, a property directly relevant to clinical decision-making when same-day reporting is required.

The titration experiments show a non-monotonic dependence on PEG concentration. At the selected operating condition, background is suppressed without a detectable effect to amplification kinetics. At higher PEG levels, positive traces display greater dispersion in their rise profiles, consistent with increased solution viscosity and altered strand-displacement dynamics. These findings suggested a narrow-optimized condition and support viewing PEG as a tunable modulator of reaction buffer rather than a universal enhancer, indicating that condition-specific optimization will be required when adapting the approach to other primer sets, enzymes, or buffer formulations.

Several limitations should be noted. The clinical panel is modest in size and includes spiked positives; therefore, matrix effects and natural variability are not fully represented. The assays were read by real-time fluorescence; performance with colorimetric or lateral-flow formats, which are relevant to near-patient testing, has not been established. Future work should expand evaluation to larger, blinded cohorts and detection targets that include etiologically related pathogens such as HSV-1/2, EBV, and CMV, and to diverse clinical matrices (Schremser et al., 2020). Integration with lyophilized reagents, simple heaters, and visual endpoints would clarify suitability for point-of-care testing.

In summary, modest PEG-induced crowding provides a low-complexity means to suppress nonspecific LAMP reactions while maintaining on-target amplification efficiency. Under an optimized condition, the assay combines a lower detection limit, faster time-to-result, and qualitative agreement with PCR on the evaluated panel. These attributes are directly relevant to VZV diagnostics, where earlier and more reliable calls can reduce reflex testing and support timely clinical decisions, including in near-patient and resource-limited settings. Because the modification acts by tuning the reaction microenvironment rather than altering primers or instrumentation, the approach is readily portable to other LAMP targets. These findings establish PEG-LAMP as a feasible, low-complexity enhancement that delivers PCR-level qualitative accuracy with faster readout, advancing the practical deployment of isothermal diagnostics for VZV in both routine and resource-limited settings.

## Data Availability

All data generated or analyzed during this study are included in this published article.
